# Supplementary material for: Monitoring Immune Modulation in Season Population: Identifying Effects and Markers Related to Apis mellifera ligustica Honey Bee Health
Source: Biomolecules. 2023 Dec 22;14(1):19. doi: 10.3390/biom14010019 (PMC10813216; doi:10.3390/biom14010019)
Supplement: Supplementary file 1 [file biomolecules-14-00019-s001.zip › biomolecules-2743289-supplementary.pdf]

Supplementary material

Monitoring Immune Modulation in Season Population: Identifying Effects and Markers Related to *Apis mellifera ligustica* Honey Bee Health

Olga Frunze[1,2†], Hyunjee Kim[1,2†], Byung-ju Kim[1,2], Jeong-Hyeon Lee[1,2], Mustafa Bilal[1,2] and Hyung-Wook Kwon[1,2*]

[1]Department of Life Sciences , Incheon National University, 119 Academy-ro, Yeonsu-gu, Incheon 22012, Republic of Korea; frunzeon@gmail.com (O.F.); beamed79@hanmail.net (H.K.); bjk@inu.ac.kr (B.-j.K.); jhl2532@naver.com (J.-H.L.); tuci4ka2006@gmail.com (M.B.)

[2]Convergence Research Center for Insect Vectors (CRCIV), Incheon National University, 119 Academy-ro, Yeonsu-gu, Incheon 22012, Republic of Korea

*Correspondence: hwkwon@inu.ac.kr; Tel.: +82-10-3379-6727

†These authors contributed equally to this work.

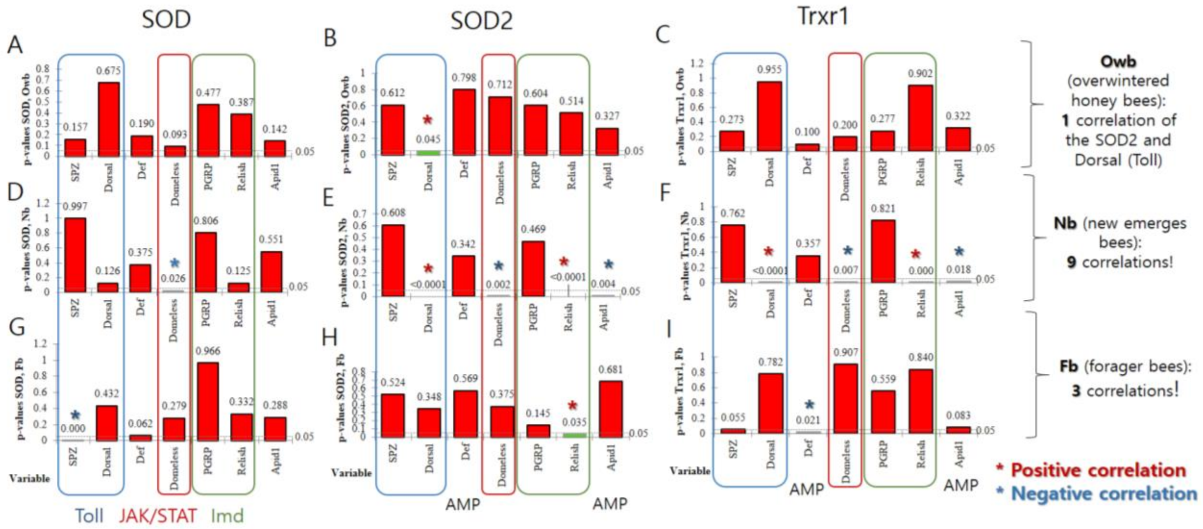

Figure S1. Correlation P-value of the continuous variables ROS enzymes (*SOD, SOD2*, and *Trxr1*) against the quantitative variables of immune path genes (X-axis) (Pearson's Phi method) in honey bee winter (Owb: A, B, and C) and spring populations (Nb: D, E, and F; Fb: G, H, and I). Owb - overwintered bees of the winter population; Nb – newly emerge bees of the spring population; Fb – forager bees of the spring population.

Table S1. Correlation matrix of Fb (forager honey bees)

Correlation matrix (Pearson) / Group Fb:										
Variables	SOD	SOD2	Trxr1	SPZ	Dorsal	Def	Domeless	PGRP	Relish	Apid1
SOD	**1**	-0.304	**-0.679**	**-0.945**	-0.301	0.643	-0.405	-0.017	-0.366	0.399
SOD2	-0.304	**1**	0.089	0.245	0.355	0.221	0.337	-0.527	**0.702**	0.160
Trxr1	**-0.679**	0.089	**1**	0.657	0.108	**-0.744**	0.046	-0.226	0.079	-0.607
SPZ	**-0.945**	0.245	0.657	**1**	0.507	-0.619	0.578	0.104	0.528	-0.249
Dorsal	-0.301	0.355	0.108	0.507	**1**	0.195	**0.894**	-0.176	**0.847**	0.626
Def	0.643	0.221	**-0.744**	-0.619	0.195	**1**	0.212	-0.356	0.190	**0.856**
Domeless	-0.405	0.337	0.046	0.578	**0.894**	0.212	**1**	-0.038	**0.849**	0.604
PGRP	-0.017	-0.527	-0.226	0.104	-0.176	-0.356	-0.038	**1**	-0.200	-0.200
Relish	-0.366	**0.702**	0.079	0.528	**0.847**	0.190	**0.849**	-0.200	**1**	0.486
Apid1	0.399	0.160	-0.607	-0.249	0.626	**0.856**	0.604	-0.200	0.486	**1**
Values in bold are different from 0 with a significance level alpha=0.05										

Table S2. Correlation matrix of Nb (newly emerged honey bees)

Correlation matrix (Pearson) / Group Nb:										
Variables	SOD	SOD2	Trxr1	SPZ	Dorsal	Def	Domeless	PGRP	Relish	Apid1
SOD	**1**	0.406	0.378	0.001	0.413	-0.247	**-0.573**	0.069	0.414	-0.167
SOD2	0.406	**1**	**0.888**	-0.144	**0.996**	-0.264	**-0.728**	0.202	**0.984**	**-0.691**
Trxr1	0.378	**0.888**	**1**	-0.085	**0.901**	-0.256	**-0.667**	0.064	**0.814**	**-0.600**
SPZ	0.001	-0.144	-0.085	**1**	-0.127	**-0.687**	**0.521**	**0.687**	-0.132	0.176
Dorsal	0.413	**0.996**	**0.901**	-0.127	**1**	-0.261	**-0.740**	0.228	**0.985**	**-0.694**
Def	-0.247	-0.264	-0.256	**-0.687**	-0.261	**1**	-0.107	-0.495	-0.267	-0.087
Domeless	**-0.573**	**-0.728**	**-0.667**	**0.521**	**-0.740**	-0.107	**1**	0.164	**-0.730**	0.495
PGRP	0.069	0.202	0.064	**0.687**	0.228	-0.495	0.164	**1**	0.277	-0.123
Relish	0.414	**0.984**	**0.814**	-0.132	**0.985**	-0.267	**-0.730**	0.277	**1**	**-0.690**
Apid1	-0.167	**-0.691**	**-0.600**	0.176	**-0.694**	-0.087	0.495	-0.123	**-0.690**	**1**
Values in bold are different from 0 with a significance level alpha=0.05										

Table S3. Correlation matrix of Owb (overwintered honey bees)

Correlation matrix (Pearson) / Group Owb:										
Variables	SOD	SOD2	Trxr1	SPZ	Dorsal	Def	Domeless	PGRP	Relish	Apid1
SOD	**1**	-0.059	0.514	-0.513	-0.163	0.481	-0.593	-0.273	-0.329	-0.530
SOD2	-0.059	**1**	0.518	0.197	**0.677**	0.100	0.144	-0.201	0.251	0.370
Trxr1	0.514	0.518	**1**	-0.410	0.022	0.582	-0.471	-0.407	0.048	-0.374
SPZ	-0.513	0.197	-0.410	**1**	0.624	**-0.930**	**0.922**	**0.818**	-0.501	**0.968**
Dorsal	-0.163	**0.677**	0.022	0.624	**1**	-0.466	0.446	0.214	-0.149	**0.719**
Def	0.481	0.100	0.582	**-0.930**	-0.466	**1**	**-0.840**	**-0.817**	0.527	**-0.852**
Domeless	-0.593	0.144	-0.471	**0.922**	0.446	**-0.840**	**1**	**0.762**	-0.416	**0.868**
PGRP	-0.273	-0.201	-0.407	**0.818**	0.214	**-0.817**	**0.762**	**1**	**-0.691**	**0.702**
Relish	-0.329	0.251	0.048	-0.501	-0.149	0.527	-0.416	**-0.691**	**1**	-0.347
Apid1	-0.530	0.370	-0.374	**0.968**	**0.719**	**-0.852**	**0.868**	**0.702**	-0.347	**1**
Values in bold are different from 0 with a significance level alpha=0.05										

Table S4. Classification function of Discriminant Analysis for Fb (foragers)

Gene	Fb	Rank Fb
Intercept	-27.453	
domeless	**26.971**	**1**
spz	**3.786**	**2**
PGRP	-1.224	3
apid1	-1.078	4
Trxr1	0.323	5
relish	0.116	6
SOD2	-0.094	7
def-2	0.047	8
SOD	0.004	9
dorsal	0.000	10

Table S5. Classification function of Discriminant Analysis for Nb (newly emerged honey bees)

Gene	Nb	Rank Nb
Intercept	-38.163	
domeless	**34.250**	**1**
spz	**1.782**	**2**
apid1	-1.531	3
Trxr1	0.979	4
SOD2	-0.457	5
relish	0.390	6
PGRP	-0.248	7
def-2	0.071	8
SOD	0.004	9
dorsal	0.000	10

Table S6. Classification function of Discriminant Analysis for Owb (overwintered honey bees)

Gene	Owb	Rank Owb
Intercept	-11.187	
domeless	**8.724**	**1**
spz	**4.713**	**2**
Trxr1	0.855	3
SOD2	-0.584	4
apid1	-0.488	5
PGRP	-0.348	6
relish	0.377	7
def-2	0.041	8
SOD	0.002	9
dorsal	0.000	10
